# Effect of Isokinetic Training with Blood Flow Restriction During Rest Interval Versus Exercise on Muscle Strength, Hypertrophy, and Perception: A Pilot Study

**DOI:** 10.14789/jmj.JMJ23-0014-OA

**Published:** 2023-11-16

**Authors:** TSUKASA TANAKA, ATSUSHI KUBOTA, HAYAO OZAKI, HIROFUMI NISHIO, SHOJIRO NOZU, YUJI TAKAZAWA

**Affiliations:** 1 Graduate School of Health and Sports Science, Juntendo University, Chiba, Japan; 1 Graduate School of Health and Sports Science, Juntendo University, Chiba, Japan; 2 School of Health and Sports Science, Juntendo University, Chiba, Japan; 2 School of Health and Sports Science, Juntendo University, Chiba, Japan; 3 Institute of Health and Sports Science, Juntendo University, Chiba, Japan; 3 Institute of Health and Sports Science, Juntendo University, Chiba, Japan; 4 School of Health Science, Tokai Gakuen University, Aichi, Japan; 4 School of Health Science, Tokai Gakuen University, Aichi, Japan; 5 Department of Sports Medicine, Faculty of Medicine, Juntendo University, Tokyo, Japan; 5 Department of Sports Medicine, Faculty of Medicine, Juntendo University, Tokyo, Japan

**Keywords:** vascular occlusion, ischemic, blood flow restriction, pain, discomfort

## Abstract

**Objectives:**

This study aimed to determine the effects of high-intensity isokinetic training with blood flow restriction during rest interval between set (rBFR) versus during exercise (eBFR) on muscle hypertrophy and increasing muscle strength and determine whether BFR-induced exercise pain is suppressed by rBFR.

**Materials and Methods:**

Fourteen arms (7 participants) were recruited for the study. We conducted the following interventions for each arm: eBFR (n=4), rBFR (n=5), and exercise only (CON, n=5). The participants performed elbow flexion training with a BIODEX device twice weekly for 8 weeks. This study training consisted of total four sets; each was performed until <50% peak torque was achieved twice consecutively. BFR pressure was set at 120 mmHg. Elbow flexor peak torque during concentric contraction (CC), isometric contraction (IM), and muscle cross-sectional area (CSA) were measured before and after the intervention. Numerical rating scale scores used to assess pain during exercise were determined during training.

**Results:**

Peak torque at the CC increased in the rBFR (p<0.05) and IM increased in the rBFR and CON (p<0.05), while CSA increased in the rBFR and CON (p<0.001). The pain during exercise was severe in the eBFR and moderate in the rBFR and CON.

**Conclusions:**

This study's showed that high-intensity isokinetic training with rBFR did not have a synergistic effect on increasing muscle strength and muscle size. Additionally, high-intensity isokinetic training with BFR when it may be best not to perform it during exercise, because it was induces severe pain and may inhibit increases in muscle strength.

## Introduction

Isokinetic training has the advantage of being able to measure muscle strength at any angle of the range of motion and has been used primarily in rehabilitation^1, 2)^. Therefore, isokinetic training can be performed at maximum effort in participants who have difficulty performing high-intensity exercise (e.g., postoperative patients and the elderly), and can improve muscle strength and muscle size^3, 4)^. However, there are many cases of muscle weakness and persistent pain in the affected area due to muscle atrophy. In such cases, low-intensity exercise was chosen because maximal effort exercise is difficult.

Many recent studies have shown that blood flow restriction training (BFRT) is useful for rehabilitation^5, 6)^. BFRT is highly effective for muscle hypertrophy and increasing muscle strength, even using low-intensity exercises, and useful for many people^7, 8)^. BFRT is a simple method in which blood flow is restricted by a cuff attached to a limb. However, BFRT induces sever pain and discomfort during exercise than traditional training without it^9)^. This problem negatively impacts participant motivation; thus, better options are needed.

Previous studies, pain during exercise with BFRT was due to excessive pressure caused by muscle contraction and cuff placement^9, 10)^. Therefore, an alternative to BFR during exercise (eBFR) is required. The effects of exercise and BFR (e.g., interval rest) on pain during exercise, muscle hypertrophy, and increased muscle strength were recently investigated. The rating of perceived exertion and pain were lower for BFR performed during rest interval between set (rBFR) than those during eBFR, and muscle size and muscle function were comparable to eBFR and rBFR^11-13)^. High-intensity exercise with eBFR has no training effects^14)^, but high-intensity exercise with rBFR increases metabolic stress^15)^. In brief, training with rBFR may increase muscle hypertrophy and function while reducing pain.

Previous study combined rBFR with high-intensity exercise have shown no synergistic effects on increasing muscle strength or muscle size^12)^. However, the exercise task used leg curls, and the effect of combining rBFR with isokinetic training with maximal effort is unknown. We expected that isokinetic training with maximal effort combining rBFR would be more effective than eBFR and non-BFR at improving muscle hypertrophy and increasing muscle strength. Furthermore, the usefulness of rBFR has only recently been reported, and consensus is lacking about the most effective timing for BFR. This study aimed to determine the effects of high-intensity isokinetic training with rBFR and eBFR on muscle hypertrophy, muscle strength, and exercise pain caused by BFR was suppressed by rBFR.

## Materials and Methods

### Participants

This study assessed seven healthy men (mean ± standard deviation [SD]: age, 23.6 ± 1.0 years; height, 173.5 ± 5.5 cm; weight, 66.4 ± 4.7 kg). The exclusion criteria were a history of injury to the upper arm and habitual training. The participants were instructed not to perform upper-arm training until after the study. The purpose, methods, procedures, risks, and compensation for this study were explained to the participants verbally and in writing, and all participants gave informed consent. The study was conducted in accordance with the guidelines of the Declaration of Helsinki and approved by the Ethics Committee for Human Experiments of Juntendo University, Japan (no. 30-28).

### Design

**Figure 1:**
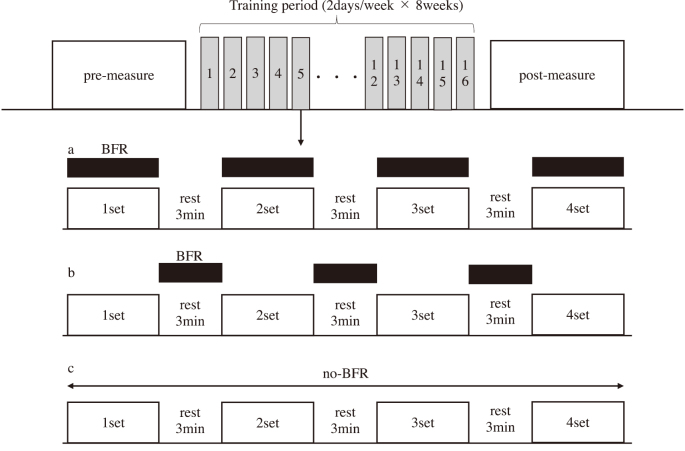
Overview of study design and training protocol. a: blood flow restriction during exercise (eBFR), b: blood flow restriction during rest interval between set (rBFR), c: Training only (CON). The black squares is blood flow restriction (BFR).

Figure 1 showed an overview of the study design. The participants visited the laboratory 3-5 times for pre-measurement of elbow flexor strength and cross-sectional area (CSA) before the start of training. After the pre-measurement is completed, we conducted the following interventions to each arm (n = 14) subjected to the following interventions: eBFR (n = 4); rBFR (n = 5); and training only (CON: n = 5). The assignment of each condition was determined randomly that not both arms of the same participant would have the same condition. The training involved elbow flexor exercises twice weekly for 8 weeks. Pain during exercise was assessed using the numerical rating scale (NRS). Elbow flexor strength and CSA were measured before and after 8 weeks of training.

### Training protocol

Figure 1 showed an overview of the training protocol. The training included elbow flexor exercises using a BIODEX device (BIODEX medical system, Shirley, NY, USA). The participants performed an elbow flexion exercise at 60°/sec under concentric contraction (CC) with a ROM of 120° (full extension = 0°). Training repetitions were performed at maximal effort. Training consisted of four sets with a 3-min rest interval between sets. This rest time was determined from the previous study^16)^ on training rest time and our preliminary experiment. The standard for the end of each set were defined as until the participant could not achieve >50% of the peak torque of the CC pre-measurement twice consecutive (= one set). This was based on previous study that evaluated muscle fatigue after exercise with BFR training^17)^. The training was performed twice weekly for 8 weeks.

### Blood flow restriction

BFR was induced on the proximal upper arm using a cuff (width, 77 mm; length, 770 mm; MIZUHO, Tokyo, Japan) at a pressure of 120 mmHg. This pressure volume was 100% of the systolic blood pressure in Japanese individuals determined based on previous study results^18)^. The rBFR group underwent BFR training at rest, whereas the eBFR group underwent BFR training during exercise.

### Muscle strength

Before and after training, changes in elbow flexor strength were measured using CC and isometric contraction (IM) with a BIODEX device. The measurements were performed with the participants in a sitting position with two belts fixed on the shoulder and one on the abdomen. IM contraction measurements were made with the elbow in a 90° flexed position. The participants performed 3-5 reps warm up at sub maximum before the true measurement.

### Cross-sectional area

Before and after training, changes in the CSA of the biceps were measured on magnetic resonance imaging (E-scan XQ; 0.2T, ESAOTE, Genoa, Italy). Measurements were made at 60% distally between the lateral epicondyle and the acromial process using T1-weighted magnetic resonance imaging with the spin-echo method (slice number, 28; slice width, 5 mm). Osirix open software (version 10.0.0; Pixmeo Sàrl, Bernex, Switzerland) was used to calculate muscle CSA. The measurements were performed twice before the training session, and the coefficient of variation was confirmed as being within 2%.

### NRS scores

NRS score is a subjective score on an 11-point scale from 0 to 10. This study, subjective pain during exercise were used to assess using the NRS and the degree of arm pain was as follows; (0 = no pain; 1-3 = mild pain; 4-6 = moderate pain; 7-9 = severe pain; and 10 = extreme pain). The NRS scores were evaluated immediately after each set.

### Statistical analysis

All data are shown as median (min-max). The analysis of muscle strength and CSA before and after training was performed using the Wilcoxon signed-rank test (pre-post), while the intergroup analysis was performed using the Kruskal-Wallis test. NRS and exercise repetition results were analyzed using Friedman’s test for each set, and the NRS and exercise repetitions of the intergroup analysis were examined using the Kruskal-Wallis test. Effect sizes were calculated from the [r = Z√N ] formula. Effect sizes were rated as follows: <0.1 small, <0.3 medium, <0.50 large. The statistical analyses were performed using SPSS Statistics (version 22.0; IBM, Armonk, NY, USA), and the significance level was set at p < 0.05.

## Results

**Figure 2:**
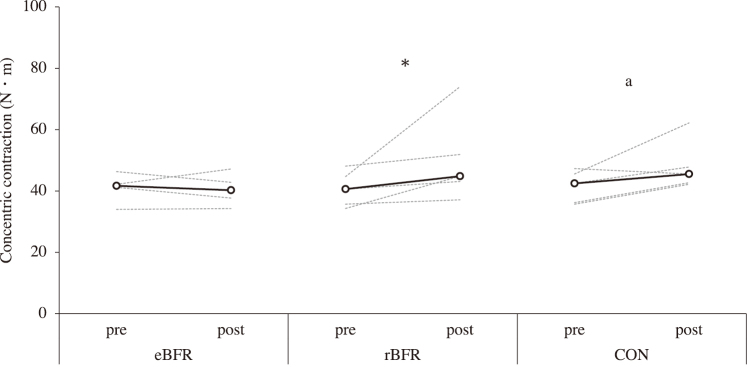
Elbow flexor isometric contraction strength after training to 8 weeks. eBFR: blood flow restriction during exercise, rBFR: blood flow restriction during rest interval between set, CON: training only. Black line shows the median value, and gray line showed the personal values. * = p<0.05, a = p<0.10.

**Figure 3:**
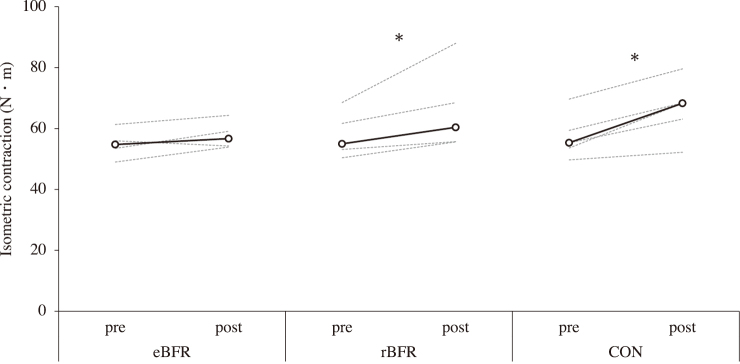
Elbow flexor concentric contraction strength after training to 8 weeks. eBFR: blood flow restriction during exercise, rBFR: blood flow restriction during rest interval between set, CON: training only. Black line shows the median value, and gray line showed the personal values. * = p<0.05.

**Figure 4:**
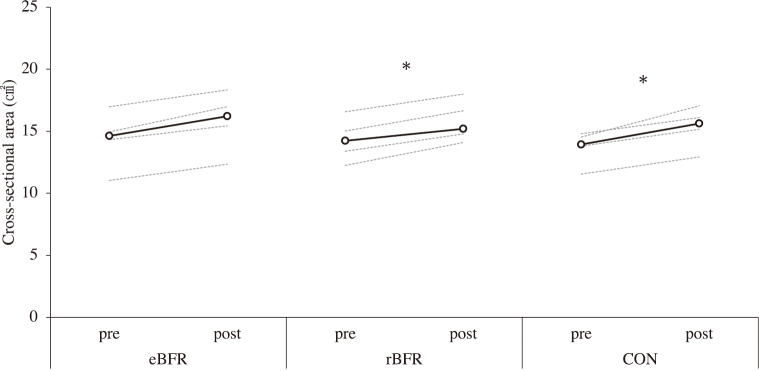
Cross-sectional area of the biceps after training to 8 weeks. eBFR: blood flow restriction during exercise, rBFR: blood flow restriction during rest interval between set, CON: training only. Black line shows the median value, and gray line showed the personal values. * = p<0.05.

Figure 2 shows the changes in CC after training. The peak torque at the CC increased after the intervention in the rBFR (pre: 40.6 N・m [34.3-48.1], post: 44.8 N・m [37.1-74.0]; p = 0.043; r = 0.64) while a marginally significant increase was noted in the CON (pre: 42.5 N・m [35.7-47.3], post: 45.5 N・m [42.2-62.2]; p < 0.10; r = 0.64) versus no increase after the intervention in the eBFR (pre: 41.7 N・m [34.0-46.3], post: 40.3 N・m [34.3-47.2]; r = 0.00). Figure 3 shows the changes in IM after training. The peak torque at the IM increased after the intervention in the rBFR (pre: 55.0 N・m [50.4-68.5], post: 60.4 N・m [55.6-88.0]; p = 0.043; r = 0.64) and CON (pre: 55.3 N・m [49.7-69.7], post: 68.3 N・m [52.2-79.6]; p = 0.011; r = 0.64) groups but did not increase after the intervention in the eBFR (pre: 54.8 N・m [49.4-64.3], post: 56.7 N・m [53.9-64.3]; r = 0.52). Figure 4 shows the changes in CSA after training. The CSA increased after the intervention in the rBFR (pre: 14.2 cm^2^ [12.2-16.1], post: 15.2 cm^2^ [14.1-18.0]; p = 0.005; r = 0.64) and CON (pre: 13.9 cm^2^ [11.5-14.8], post: 15.6 cm^2^ [12.9-17.0]; p = 0.002; r = 0.64) but did not increase after the intervention in the eBFR (pre: 14.6 cm^2^ [11.0-17.0], post: 16.2 cm^2^ [12.3-18.3]; r = 0.65). All measurement items did not differ significantly among the conditions.

Table 1 shows the changes in exercise repetition, torque, and pain during exercise. The exercise repetitions decreased in three sets for eBFR and CON and in 4 set for rBFR and CON versus 1 set (p < 0.05). The exercise repetitions were lower for the eBFR than CON for all sets (p < 0.05), while the rBFR was marginally lower in 1 set (p = 0.67). The torque during exercise decreased after three sets in the rBFR (vs. 1 set; p = 0.042) and CON (vs. 2 set; p = 0.029). Also, there were no significant differences between conditions in the total repetitions for all training sessions (eBFR: 1016 rep [779-1233], rBFR: 1130 rep [960-2213], CON: 2014 rep [1233-3326].

**Table 1:** Exercise repetitions, Torque, pain during exercise

			1set	2set	3set	4set
Repetition	eBFR (n=4)		14 (10-21)	12 (9-18)	11 (9-17)^＊^	11 (9-18)
	rBFR (n=5)		27 (22-61)^b^	19 (12-31)	18 (11-29)	16 (11-26)^＊^
	CON (n=5)		35 (22-63)^#^	30 (20-59)^#^	29 (17-47)^＊#^	30 (17-37)^＊#^
Torque (N・m)	eBFR		31.5 (29.8-33.3)	33.0 (32.8-33.3)	32.6(31.3-33.0)	31.6 (30.3-32.3)
	rBFR		41.0 (31.0-45.0)	32.8 (25.5-42.0)	31.5 (25.0-39.8)^＊^	30.3 (25.5-44.5)
	CON		39.0 (36.0-41.5)	38.3 (36.3-40.3)	36.5 (33.5-38.5)^†^	37.0 (33.8-40.0)
Pain	eBFR		6.6 (5.3-8.4)	7.4 (5.4-9.0)	7.9 (5.8-8.6)	7.8 (5.7-8.4)
	rBFR		5.6 (3.0-7.0)	5.9 (3.6-6.8)	6.3 (3.9-7.3)	6.5 (4.0-7.9)
	CON		5.4 (3.0-6.8)	5.8 (3.1-6.9)	6.4 (3.1-7.1)	6.7 (3.3-7.1)^＊^

Data were median (min-max). Blood flow restriction during exercise (eBFR), Blood flow restriction during rest interval between set (rBFR), Training only (CON). ＊ = p<0.05 (vs. pre), † = p<0.05 (vs. 2set), b = p<0.10, # = p<0.05 (vs. eBFR).

Pain during exercise was not significantly different among the conditions for all sets. However, pain during exercise was increased for 4 set versus 1 set in the CON group (p = 0.004). Table 2 shows the number of respondents in pain intensity at the final training session.

**Table 2:** Number of Numerical Rating Scale respondents

		Numerical Rating Scale score										
		0	1	2	3	4	5	6	7	8	9	10
1set	eBFR (n=4)	-	-	-	-	1	1	-	-	-	2	-
	rBFR (n=5)	-	-	-	-	2	-	3	-	-	-	-
	CON (n=5)	-	-	-	1	-	3	-	1	-	-	-
4set	eBFR	-	-	-	-	1	-	-	2	-	-	1
	rBFR	-	-	-	1	-	2	1	-	1	-	-
	CON	-	-	-	-	1	1	2	-	-	-	1

The numbers in the table show the number of respondents, and this data were the final training session. eBFR: Blood flow restriction during exercise, rBFR: Blood flow restriction during rest interval between set, CON: training only. Scale were follows: 0; no pain, 1-3; mild pain, 4-6; moderate pain, 7-9; severe pain, 10; extreme pain.

## Discussion

The study investigated isokinetic training with maximum effort for 8 weeks in eBFR, rBFR, and CON. These results showed that elbow flexor strength and muscle CSA increased with rBFR and CON. However, there were no statistically significant in elbow flexor strength and muscle CSA between the conditions, and the pain intensity was “severe pain” in eBFR, and “moderate pain” in rBFR and CON between the conditions. Our findings suggest that maximal effort of isokinetic training with eBFR inhibits hypertrophy and increase muscle strength. In contrast, rBFR suggested equally effects for CON, and does not exacerbate pain during exercise.

This study found no significant differences in the increase in muscle strength among the conditions. However, increasing CC was induced in the rBFR and CON, and rBFR may be more effective than eBFR as the effect size was higher in the rBFR (< 0.50 large) and CON (< 0.50 large) than the eBFR (< 0.10 small). In a previous study, training volume was important for muscle hypertrophy^19)^, and muscle size and neuromuscular activation improved muscle strength^20)^. Therefore, high- intensity isokinetic training with the eBFR does not provide the required training volume or intensity. Muscle strength should decrease with accumulated fatigue, but only eBFR did not decrease. Therefore, we speculated that eBFR did not have a training effect because it did not provide the required torque or training volume during exercise. Furthermore, a previous study used resistance training, which offered varied results as a result of the different exercise methods. A previous study that combined BIODEX exercises with BFR reported increased muscle strength^21)^. However, the participants were athletes and the effect greater, with a relatively high-speed CC of 300°/sec. Consequently, varied results might be attributed to changes in the participants examined as well as the use of a CC of 60°/sec, a slower contraction speed than those in previous studies. Additionally, BFR alone effectively prevented disuse muscular atrophy and muscle weakness^22, 23)^. Therefore, rBFR may be even more effective in postoperative patients with muscle atrophy who require muscle function recovery.

Muscle perception did not differ significantly among conditions; however, moderate pain was reported for rBFR and CON, whereas severe pain was reported for eBFR. In addition, table 2 also shows that the number of participants who complained of severe pain was 2 to 3, but only 1-participant each complained of rBFR and CON. This indicates that the pain was not excessive in the rBFR group. In addition, ischemia-reperfusion numbness is caused by stimulation of the sensitization of transient receptor potential ankyrin 1 with active oxygen generated by reperfusion^24)^. Therefore, if there is an effect of ischemia-reperfusion, rBFR is expected to be more painful during exercise. Furthermore, BFR during exercise and rest (continue BFR [cBFR]) reportedly increases pain, in which cases intermittent BFR has been recommended^25)^. However, previous studies of rBFR versus cBFR (345 sec/session) with rBFR (30 sec/session)^11)^. However, this study considered that BFR did not induce excessive pain because it was performed for a short time of 60-180 sec/session. In addition, blood flow is inhibited by intramuscular pressure, even at a low muscle contraction of 20-30% of one repetition maximum^26)^, and the perception of pressure pain is influenced by the external stimulus intensity and stimulation area^27, 28)^. Based on these results, muscle contraction and cuff pressure overlapped, thereby contributing to the increased pain. Accordingly, the method of separating exercise from BFR is expected to effectively improve pain.

The present study has some limitations. First, the BFR pressure used was 120 mmHg, and different BFR pressures may have produced different results. Previous studies reported that the training effects of BFR are equal regardless of the use of high or low pressure^29, 30)^, however, these results are for eBFR. In the future, the effect of different volumes of pressure on rBFR should be examined. Second, the training in this experiment involved isokinetic resistance training using a BIODEX device, and each repetition was performed at the maximum output, representing high-intensity training. In previous study, BFR was not useful in high-intensity training^14)^. However, studies of athletes indicated that BFR is more effective, particularly during intense training^21, 31)^. Furthermore, there are several components of the training impact, and the current study focused on the influence on muscular hypertrophy and strength. Future studies of rBFR that evaluate items and training intensities may be useful. Third, this was a pilot study with a small number of participants. Future studies with larger groups of people are needed to determine the usefulness of rBFR. However, few studies have investigated the long-term interventions for rBFR. In particular, this is the first study to examine it in isokinetic training. We believe that these results will inform future studies of the usefulness of rBFR.

In conclusion, this study's showed that high- intensity isokinetic training with rBFR did not have a synergistic effect on increasing muscle strength and muscle size. Additionally, high-intensity isokinetic training combined with BFR when it may be best not to perform it during exercise, because it was induces severe pain and may inhibit increases in muscle strength.

## Funding

This study was supported by the Juntendo Administration for Sports, Health and Medical Sciences.

## Author contributions

TT, AK and HO conceived of and designed the study. TT conducted the experiments and drafted the manuscript. AK, HO, HN, SN, and YT revised the manuscript and contributed to the data interpretation. All authors approved the version to be published.

## Conflicts of interest statement

The authors have no competing interests to declare that are relevant to the content of this article and did not receive support from any organization for the submitted work.
